# Genome-Wide DNA Methylation Analysis of Chinese Patients with Systemic Lupus Erythematosus Identified Hypomethylation in Genes Related to the Type I Interferon Pathway

**DOI:** 10.1371/journal.pone.0169553

**Published:** 2017-01-13

**Authors:** Kit San Yeung, Brian Hon-Yin Chung, Sanaa Choufani, Mo Yin Mok, Wai Lap Wong, Christopher Chun Yu Mak, Wanling Yang, Pamela Pui Wah Lee, Wilfred Hing Sang Wong, Yi-an Chen, Daria Grafodatskaya, Raymond Woon Sing Wong, Chak Sing Lau, Daniel Tak Mao Chan, Rosanna Weksberg, Yu-Lung Lau

**Affiliations:** 1 Department of Paediatrics and Adolescent Medicine, Li Ka Shing Faculty of Medicine, The University of Hong Kong, Hong Kong, China; 2 Genetics and Genome Biology Program, The Hospital for Sick Children Research Institute, Toronto, Canada; 3 Department of Medicine, Li Ka Shing Faculty of Medicine, The University of Hong Kong, Hong Kong, China; 4 Department of Biomedical Sciences, The City University of Hong Kong, Hong Kong, China; 5 Division of Clinical and Metabolic Genetics, The Hospital for Sick Children, Toronto, Ontario, Canada; 6 Institute of Medical Science and Department of Pediatrics, University of Toronto, Toronto, Ontario, Canada; Instituto Nacional de Ciencias Medicas y Nutricion Salvador Zubiran, MEXICO

## Abstract

**Background:**

Epigenetic variants have been shown in recent studies to be important contributors to the pathogenesis of systemic lupus erythematosus (SLE). Here, we report a 2-step study of discovery followed by replication to identify DNA methylation alterations associated with SLE in a Chinese population. Using a genome-wide DNA methylation microarray, the Illumina Infinium HumanMethylation450 BeadChip, we compared the methylation levels of CpG sites in DNA extracted from white blood cells from 12 female Chinese SLE patients and 10 healthy female controls.

**Results:**

We identified 36 CpG sites with differential loss of DNA methylation and 8 CpG sites with differential gain of DNA methylation, representing 25 genes and 7 genes, respectively. Surprisingly, 42% of the hypomethylated CpG sites were located in CpG shores, which indicated the functional importance of the loss of DNA methylation. Microarray results were replicated in another cohort of 100 SLE patients and 100 healthy controls by performing bisulfite pyrosequencing of four hypomethylated genes, *MX1*, *IFI44L*, *NLRC5* and *PLSCR1*. In addition, loss of DNA methylation in these genes was associated with an increase in mRNA expression. Gene ontology analysis revealed that the hypomethylated genes identified in the microarray study were overrepresented in the type I interferon pathway, which has long been implicated in the pathogenesis of SLE.

**Conclusion:**

Our epigenetic findings further support the importance of the type I interferon pathway in SLE pathogenesis. Moreover, we showed that the DNA methylation signatures of SLE can be defined in unfractionated white blood cells.

## Introduction

Systemic lupus erythematosus (SLE) is a multi-system autoimmune disease with significant morbidity and mortality. The prevalence of SLE is higher in Asians (58.8/100,000) than in Caucasians (20.7/100,000) [1, 2]. SLE is characterized by abnormal T-cell and B-cell signaling, with auto-antibodies against nuclear antigens such as DNA, histones and nucleosomes [3, 4]. Genome-wide association studies have shown that the development of SLE is associated with variants in multiple genes, such as *PXK*, *ITGAM*, *CD80* and *CDKN1B* [5–7].

Apart from genetic factors, environmental factors such as ultraviolet B radiation, viral infections and drugs such as hydralazine or procainamide can lead to SLE [8–10]. In particular, environmental factors exert their effect through DNA methylation changes [11]. DNA methylation usually occurs on cytosine residues of cytosine-guanosine dinucleotides (CpG), and this modification can act as a transcriptional regulator. Methylation of CpG sites in the promoter region usually causes gene silencing [12]. In addition, CpG island shores (CpG shores) are defined as regions that are 0–2 kb away from a CpG island and are associated with most tissue-specific differential DNA methylation, rather than CpG islands, and the DNA methylation level in CpG shores is inversely related to gene expression [13].

A number of observations in T cells from patients with SLE have demonstrated the targeted loss of DNA methylation and overexpression of specific genes relevant to autoimmunity, leading to autoreactivity, excessive B-cell stimulation and cytotoxicity. The first evidence that lupus-associated drugs such as procainamide and hydralazine could inhibit DNA methylation was reported in 1988 [9]. It was later demonstrated that hypomethylation of the CpG sites within the regulatory sequence of *ITGAL* was observed in T cells from individuals who were treated with lupus-associated drugs, resulting in over-expression of CD11a [14]. This hypomethylation was also observed in T cells of SLE patients. CD11a strengthens the adhesion of T cells to other immune cells and thereby likely contributes to autoreactivity. Later, it was found that the promoter region of *PRF1* is hypomethylated in CD4+ T cells from lupus patients, leading to over-expression of perforin, which is a cytotoxic molecule of natural killer cells and cytotoxic T cells [15]. The promoter region of *CD70* was also found to be hypomethylated in SLE patients, leading to over-expression of CD70. This phenomenon was also observed in cells treated with DNA methylation inhibitors [16]. Lastly, the X-linked gene *TNFSF5*, encoding the CD40 ligand, which is important in T cell-dependent B-cell stimulation, is also hypomethylated and over-expressed in T cells of lupus patients [17].

In addition to targeted studies of DNA hypomethylation in gene promoter regions, several studies have investigated the genome-wide DNA methylation status in SLE patients. These studies utilized different versions of the Illumina microarray platform and included SLE patients of mixed ethnicity. Although these studies showed hypomethylation of genes, they were enriched in different biological pathways. For example, with the use of the HumanMethylation27 BeadChip, Jeffries et al. found hypomethylated genes that were related to growth of cells, apoptosis, developmental tissue processes and cell activation [18], and Ho et al. identified genes involved in cellular movement and immune cell trafficking [19]. However, more recent studies utilizing the HumanMethylation450 BeadChip (450k microarray) demonstrated that genes in the interferon pathway were persistently hypomethylated in various specific blood cell lineages [20–22].

Since DNA methylation can be affected by single nucleotide polymorphisms as well as the ethnic background of individuals [23], we undertook the first study of genome-wide DNA methylation changes in SLE patients of Chinese ethnicity using the 450k microarray, with the aim of identifying DNA methylation changes specific to Chinese ethnicity, where the prevalence of SLE is higher than in other populations. In fact, our findings in Chinese SLE patients replicated the results in previous studies indicating that hypomethylation occurred in genes related to the type I interferon pathway, which has long been implicated in the pathogenesis of SLE [24].

## Materials and Methods

### Patient selection and healthy controls

The SLE patients who participated in this study were all female and self-reported to be of Chinese ethnicity living in Hong Kong. These patients all met the criteria of the American College of Rheumatology for SLE diagnosis [25] and were over 16 years old at the time of disease onset. One additional requirement for SLE patients recruited in the microarray study was that patients within 4 years of diagnosis were selected to minimize the effect of pharmacologic treatment on DNA methylation patterns, as per Javierre et al. [26]. However, patients recruited for validation and replication by bisulfite pyrosequencing and mRNA expression analysis did not necessarily fulfill this additional requirement due to the limited number of patients. Each SLE patient was compared to sex-, age- and ethnicity-matched controls. Blood samples from healthy controls were obtained from the Hong Kong Red Cross, and these participants were self-reported to be healthy without chronic diseases. Written consent was obtained and kept in the patients’ clinical charts, which were stored by the corresponding hospitals from which the patients were recruited. This study was approved by the Institutional Review Board of the University of Hong Kong/Hospital Authority Hong Kong West Cluster (IRB Ref: UW10-380).

### Whole genome DNA methylation microarray

Twelve patients with SLE and 12 healthy controls were studied using the HumanMethylation450 BeadChip (Illumina) for genome-wide DNA methylation analysis. The clinical information of the 12 patients with SLE can be found in S1 Table. The microarray targets ~485,000 CpG sites spanning 30,000 genes, covering 99% of RefSeq genes. It provides broad coverage throughout gene regions including 1.5 kb or 2 kb upstream of the transcription start site and the five prime untranslated region (5’UTR), first exon, gene body and three prime untranslated region (3’ UTR), as well as CpG islands and the surrounding shelves and shores, for a comprehensive view of methylation levels. CpG island refers to a region of at least 2 kb with a CG content of greater than 50%, a CpG island shore (CpG shore) is the region 0 to 2 kb away from the CpG island, a CpG island shelf (CpG shelf) is the region 2 to 4 kb away from the CpG island, and regions that do not belong to either a CpG island, CpG shore or CpG shelf are referred to as non-islands. DNA was extracted from whole blood using the FlexiGene DNA Kit according to the manufacturer’s protocol (Qiagen). DNA samples were then bisulfite-converted using the EpiTect Bisulfite Kit according to the manufacturer’s protocol (Qiagen). Bisulfite-converted DNA was labeled, hybridized and scanned at the Centre for Applied Genomics at The Hospital for Sick Children, Toronto, Canada. Intensities were normalized using Illumina’s internal normalization probes and algorithms. Beta values were derived from intensities as defined by the ratio of methylated to unmethylated probes given by β = M/ (U+M+100) and were used as a measure of the effect size. Data quality was assessed using Genome Studio (Illumina) for data quality control and exported into the R statistical environment and assessed by Illumina Methylation Analyzer package for differential methylation analysis. Loci with missing beta values and with a median detection p-value > 0.01 were filtered for further analysis. For each specific region, the Wilcoxon rank-sum test was used for group comparisons, and the false discovery rate control was used for multiple testing corrections. CpG sites with an adjusted p-value < 0.05 and a mean methylation change > |0.1| were considered differentially methylated.

To evaluate the potential effect of blood cell type composition on the identified differentially methylated CpG sites, the DNA methylation profile of our samples was compared to that of different blood fractions from a study by Reinius et al. [27], which are available from the GEO repository (series GSE35069). The DNA methylation data of Reinius et al. included whole blood, peripheral blood mononuclear cells (PBMCs) and granulocytes, as well as isolated cell populations including CD4+ T cells, CD8+ T cells, CD56+ NK cells, CD19+ B cells, and CD14+ monocytes. We compared the 60 samples (each type of blood fraction contained 6 samples) from the Reinius et al. study to our 12 SLE and 10 control samples by principal component analysis, restricting the analysis to the differentially methylated CpG sites identified in SLE.

### Bisulfite pyrosequencing

An additional cohort of 100 SLE patients and 100 healthy controls were recruited to replicate the microarray results. The DNA methylation level was measured by bisulfite pyrosequencing as described [28]. In short, pyrosequencing assays containing 2 PCR primers and 1 sequencing primer were designed to target CpG sites of interest using PyroMark Assay Design Software (Qiagen). Genomic DNA was sodium bisulfite-converted as described above for the Illumina microarray and amplified using Hot-Start Taq polymerase (Qiagen). The amplicons were analyzed on a Q24 pyrosequencer (Qiagen) as specified by the manufacturer, and the percent of methylation was quantified as the ratio of C to C+T using PyroMark Q24 Software (Qiagen). The pyrosequencing of *LINE*-1 was performed using a PyroMark Q24 CpG LINE-1 assay (Qiagen). The assay design for the four genes selected from the 450k microarray results can be found in S2 Table, and the sequences flanking the differentially methylated CpG sites are shown in S1 Fig.

### Gene ontology annotation analysis

Gene Ontology annotation analysis was performed using GeneMANIA (University of Toronto, www.genemania.org) [29]. Both hypomethylated and hypermethylated genes identified in the microarray study were input separately into GeneMANIA for co-expression analysis. GeneMANIA uses many publicly available biological databases to identify any interactions among the input gene list, such as protein and genetic interactions, pathways, co-localization and co-expression.

### Real-time PCR gene expression study

Real-time PCR was performed to quantify the expression of *MX1*, *IFI44L*, *NLRC5* and *PLSCR1*. The relative amount of mRNA from 100 healthy controls and 100 SLE patients was quantitated by real-time polymerase chain reaction. An amount of 1 μg of total RNA was reverse transcribed using High-Capacity cDNA Reverse Transcription Kits (Applied Biosystems) according to the manufacturer’s protocol. cDNA was amplified by TaqMan-based real-time PCR on an Applied Biosystems 7900HT real-time PCR system according to the instructions of the manufacturer (TaqMan Gene Expression Assay). For each sample, gene expression was normalized against the expression of an endogenous control, the glyceraldehyde-3-phosphate dehydrogenase gene.

### Statistical analysis

Unless otherwise specified, Student’s t-test was performed to assess the significance between the two groups, controls and SLE patients. A p-value < 0.05 was considered statistically significant. Analysis was performed by SPSS Statistics version 19 (IBM).

## Results

### Identification of differentially methylated CpG sites in SLE patients

**Fig 1:**
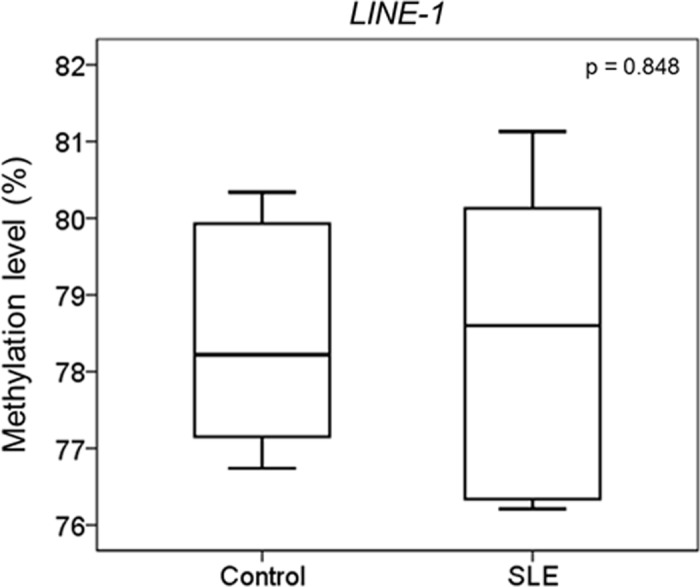
*LINE-1* methylation level measurement in control and SLE patients. Bisulfite pyrosequencing was performed for 10 controls and 12 SLE patients on three different CpG sites in *LINE-1* to determine their average methylation level. There is no significant difference between the average methylation levels of *LINE-1* in controls and SLE patients.

The genome-wide DNA methylation status of 12 female SLE patients and 12 female healthy controls was analyzed using the 450k microarray. Two control samples were excluded from further analysis as they did not meet the requirements for quality control in our study. There was no significant difference between the average β of the 485,577 CpG sites detected by the microarray in controls and SLE patients (S2 Fig). To further investigate the global DNA methylation level in SLE patients, bisulfite pyrosequencing was carried out on the long interspersed nucleotide element-1 (*LINE-1*) of the 10 controls and 12 SLE patients. The DNA methylation levels of three different CpG sites overlapping *LINE-1* were investigated, and there was no significant difference between the average methylation levels of *LINE-1* in controls and SLE patients (p = 0.848, Fig 1).

**Fig 2:**
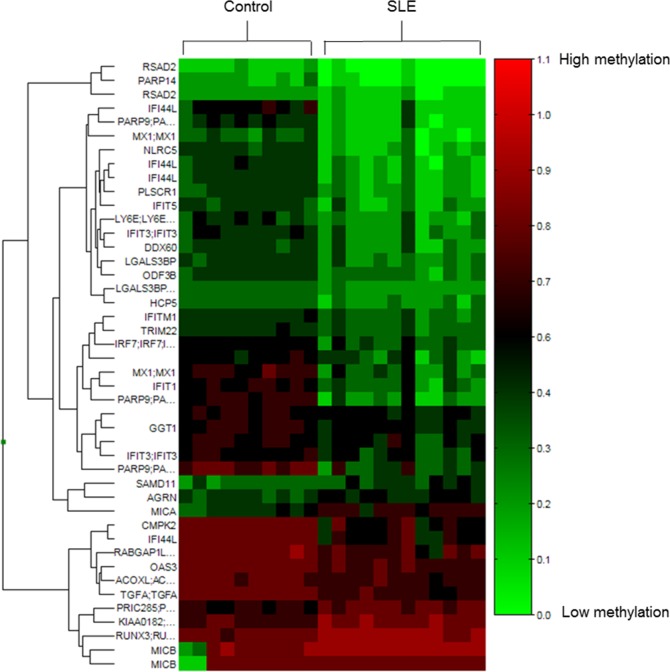
Heat map visualization of differentially methylated CpG sites. Forty-four differentially methylated CpG sites of 10 controls and 12 SLE patients are shown. CpG sites are clustered using Manhattan clustering, and the corresponding gene name of the CpG site is also shown on the left (if any). A scale is shown on the right, in which red and green correspond to a higher and a lower methylation status, respectively.

We then compared the DNA methylation status of SLE patients and controls using the Wilcoxon rank-sum test for group comparisons and applied corrections for multiple testing. Among the CpG sites detected by the microarray, there were 1012 CpG sites with an adjusted p-value < 0.05 and 1294 CpG sites with mean methylation changes > |0.1|. Only CpG sites that had both an adjusted p-value < 0.05 and a mean methylation change > |0.1| were considered differentially methylated, and 44 CpG sites fulfilled both criteria. Among the 44 differentially methylated CpG sites, 36 had loss of methylation while 8 showed gain of methylation, representing 25 and 7 genes, respectively (Fig 2). A full list of these 44 differentially methylated CpG sites can be found in S3 Table. Visual inspection of the differentially methylated sites revealed that some of the hypermethylated genes, although fulfilling the criteria of mean methylation change > 0.1 and adjusted p-value < 0.05, might not be consistently hypermethylated in SLE patients. For example, as in the case of *MICB*, differential hypermethylation was observed because two control samples had much lower average DNA methylation levels than the other 20 samples (S3 Fig). In contrast, for hypomethylated CpG sites, the differences were more consistent between the two groups.

We then examined the potential effect of blood cell type composition on the SLE methylation signature. Data on exact cell type composition are very scarce in general, and we did not have direct measurements of cell-type composition for our data samples. Instead, we used the DNA methylation profiles of 60 blood fractions from a previously published study [27]. Principal component analysis of 44 CpG sites, comparing the SLE-specific signature of our samples and samples published by Reinius et al. [27], showed a clear separation between the SLE cohort and all normal blood samples, including the different blood fractions (S4 Fig). These results clearly show that neither the normal whole blood, nor peripheral blood mononuclear cells (PBMCs), nor any of the cell subtypes isolated from such samples possess the DNA methylation profile associated with the SLE signature. Nor was the specificity of the SLE signature affected by the batch difference between our dataset and the dataset of Reinius et al. [27], demonstrating the robustness of the SLE signature and the relevance of using whole blood as a suitable clinical material for identifying SLE-specific DNA methylation signatures.

**Fig 3:**
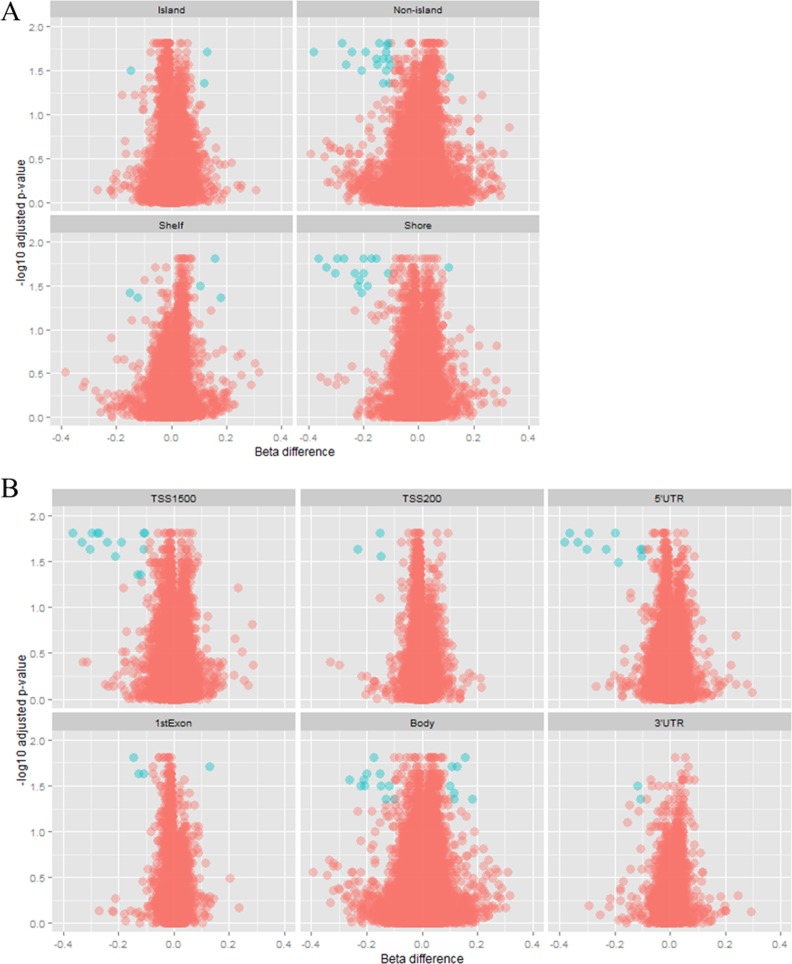
Volcano plot showing DNA methylation data according to genomic distribution. Blue spots represent CpG sites that are considered to be differentially methylated, fulfilling the requirement of a beta difference > |0.1| and log transformed adjusted p-value > 1.3, whereas red spots represent CpG sites that are not differentially methylated. (A) CpG sites are classified in relation to CpG islands. Most of the differentially methylated probes are located in shores and non-islands. (B) CpG sites are classified in relation to the gene structure, and most of the differentially methylated CpG sites are located in the 5’UTR, 1500 bp upstream of the TSS and in the gene body.

Further, we assessed the position of differentially methylated CpG sites both with respect to the CpG islands and within the gene. Of the hypomethylated CpG sites, 42% (15 of 36) were located in CpG shores. However, there was no specific pattern observed for hypermethylated CpG sites in terms of distribution with respect to CpG islands. Moreover, hypomethylated CpG sites occurred more frequently upstream of the transcription start site, in the gene body and in the 5’UTR (17, 10 and 10 sites of 36, respectively), whereas all the hypermethylated CpG sites were located in gene bodies (Fig 3).

### Replication of selected loci in an independent additional cohort

**Fig 4:**
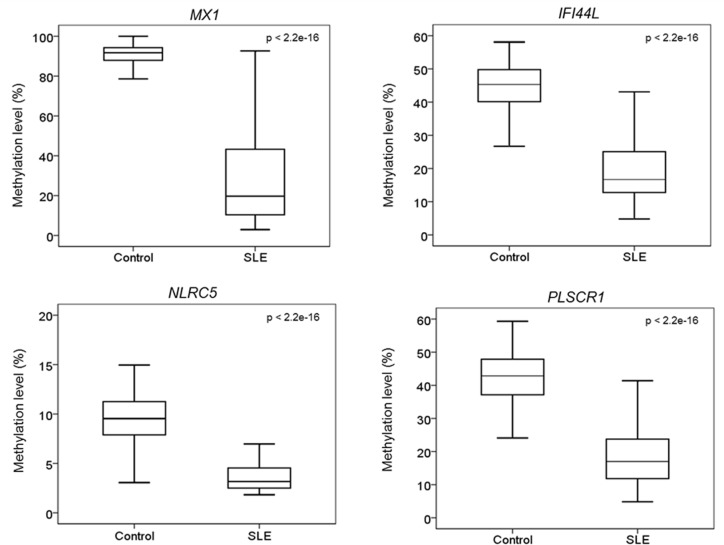
Comparison of the methylation level of four hypomethylated genes between control and SLE patients. Bisulfite pyrosequencing was carried out on four of the genes that were found to be differentially methylated in the 450k microarray and in a larger cohort of 100 SLE patients and 100 healthy controls. These genes are *MX1*, *IFI44L*, *NLRC5* and *PLSCR1*. Bisulfite pyrosequencing of all four genes confirmed the microarray findings, showing that SLE patients have a significant loss of methylation when compared to healthy controls.

To validate and replicate the differentially methylated CpG sites identified with the 450k microarray data, bisulfite pyrosequencing was carried out for four hypomethylated genes, *MX1*, *IFI44L*, *NLRC5* and *PLSCR1*, in a larger cohort of 100 controls and 100 SLE patients. The selection of these genes was based on the relevance of the genes to SLE and innate immunity, as well as the representation of probes on the microarray. For example, *IFI44L* has CpG sites with the largest degree of DNA methylation loss among all the differentially hypomethylated CpG sites identified, and genes such as *NLRC5* and *MX1* have multiple probes showing DNA methylation loss, suggesting that these genes have multiple CpG sites with DNA methylation loss. The hypomethylated CpG sites of *MX1* and *PLSCR1* selected for validation were located in CpG shores, whereas that of *NLRC5* was located in a CpG island. The tested CpG sites within these selected genes exhibited a significant loss of methylation in SLE patients when compared to controls (p < 2.2e-16, Fig 4). For *MX1* and *NLRC5*, pyrosequencing targeted an additional 6 CpG sites surrounding the one identified by microarray analysis, allowing us to expand the number of CpG sites with altered DNA methylation (S5 Fig). We found that loss of DNA methylation was not limited to the particular CpG site that was detected by microarray but could also be found in the neighboring CpG sites.

### Pathway analysis of differentially methylated genes

**Fig 5:**
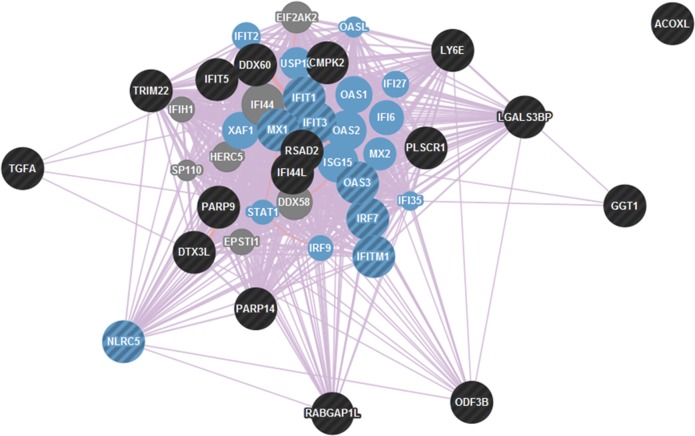
Co-expression analysis of differentially hypomethylated genes. The differentially hypomethylated genes found in the 450k microarray study were input into GeneMANIA for co-expression analysis, which revealed that they are related to type I interferon pathways, including “response to type I interferon” (FDR = 1.45e-34), “type I interferon-mediated signaling pathway” (FDR = 1.45e-34) and “cellular response to type I interferon” (FDR = 1.45e-34). Genes identified in these three co-expression pathways are overlapped with each other and are indicated in blue circles. Black circles indicate the differentially hypomethylated genes found in the 450k microarray, whereas circles with alternating black and blue stripes indicate hypomethylated genes that are overrepresented in type 1 interferon pathways.

For hypomethylated genes identified by the microarray analysis, pathway analysis revealed that these genes were highly associated with co-expression networks of type I interferon, including “response to type I interferon” (FDR = 1.45e-34), “type I interferon-mediated signaling pathway” (FDR = 1.45e-34), and “cellular response to type I interferon” (FDR = 1.45e-34) (Fig 5). There was no significant pathway enrichment found for the 7 hypermethylated genes.

### DNA methylation level and gene expression

**Fig 6:**
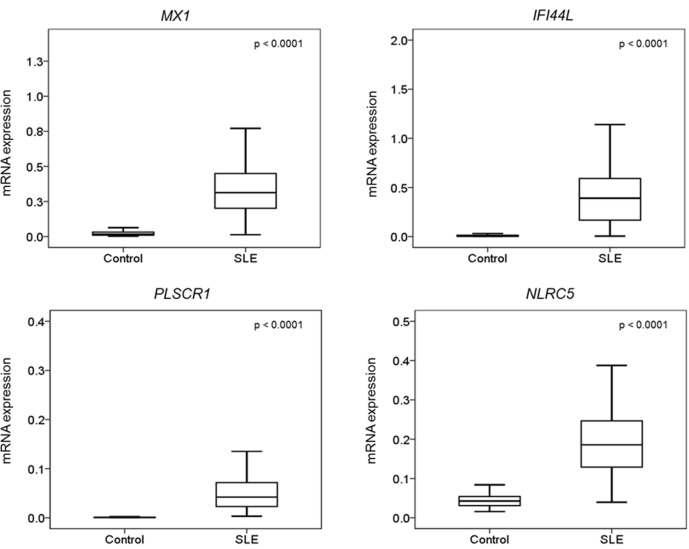
Comparison of the mRNA expression of the four validated genes between SLE patients and controls. mRNA expression level measurement of the four validated genes, *MX1*, *IFI44L*, *PLSCR1* and *NLRC5*, was carried out in a cohort of 100 controls and 100 SLE patients. The mRNA expression level of all four genes were higher in SLE patients than in controls.

To investigate the effect of DNA methylation loss on gene expression, real-time PCR analysis was performed for *MX1*, *IFI44L*, *NLRC5* and *PLSCR1* in the same cohort of individuals recruited for the bisulfite pyrosequencing analysis. The mRNA expression of these genes was significantly higher in the SLE patients than in the controls (Fig 6). Moreover, we found that the degree of methylation of the CpG sites identified in the microarray study was inversely correlated with mRNA expression (S6 Fig). The effect of DNA methylation of the CpG sites surrounding the one identified in the microarray study on mRNA expression was also investigated. In the case of *MX1* (S7 Fig), the methylation levels of the surrounding hypomethylated CpG sites (CpG2- CpG7) were compared to that of CpG1 and found to be correlated (r^2^ value = 0.66) with mRNA expression. However, in the case of *NLRC5* (S8 Fig), the methylation status of the surrounding CpG sites (CpG1- CpG6) did not correlate with the one identified by the microarray analysis. Some of the surrounding CpG sites, such as CpG3 and CpG4, did show a weak correlation with mRNA expression. The r^2^ value ranged from 0.06 to 0.26.

## Discussion

Our data contribute three new findings to the current literature. First, the epigenetic deregulation seen in Caucasian individuals with SLE can also be observed in Chinese SLE patients, although the prevalence of SLE is much higher in Chinese populations. In our study, genes exhibiting a loss of methylation in SLE patients were found to be overrepresented in type I interferon pathways. This finding is interesting but not totally surprising because the importance of type I interferon in SLE has been addressed and suggested for disease pathogenesis for several decades. For example, serum type I interferon is increased in patients with SLE [30], and the level of these proteins correlates with disease activity and severity [31]. Our initial screen for SLE candidate differentially methylated regions was performed in a small number of SLE patients (n = 12) but was then replicated in a much bigger cohort of SLE patients (n = 100). Usually, but not always, the level of DNA methylation is inversely correlated with gene expression [32, 33]. Moreover, the effect of hypomethylation on SLE patients has rarely been investigated in genome-wide DNA methylation studies. We therefore also tested the effect of the loss of methylation on gene expression in whole blood, and chose four genes with validated DNA methylation differences for further mRNA expression analysis in the same cohort of 100 patients and 100 controls. For all four genes, regardless of the location (promoter, CpG shores) of the altered CpG sites, DNA hypomethylation was associated with a statistically significant increase in mRNA expression in SLE patients. In fact, many hypomethylated genes identified in our study have already been reported in the literature to have increased expression in SLE patients. For example, *MX1*, *LY6E*, *IFIT1*, *PLSCR1* and *IRF7* were up-regulated in Caucasian SLE patients [34–36]; *MX1*, *LY6E*, and *IFIT1*, as well as *OAS1* and *OAS3*, were up-regulated in Chinese SLE patients [37–40]; and *IFITM1* was up-regulated in the platelets of Caucasian SLE patients [41]. All the above genes represent type I interferon up-regulated genes [42]. As the differentially hypomethylated genes found in our microarray study were enriched in type I interferon co-expression pathways, our study further supports the importance of type I interferon in SLE in the Chinese population. Although Asians have a higher prevalence of SLE than Caucasians, the same hypomethylation signature can be identified, therefore such hypomethylation is thought to represent the epigenetic predisposition to SLE that is independent of ethnicity. To investigate when this gene-specific hypomethylation occurs, it would be interesting to carry out long term follow-up studies of people at high risk of SLE (e.g., first-degree relatives of individuals with SLE) to see if they also develop hypomethylation of type I interferon-related genes prior to the onset of SLE.

Second, such epigenetic deregulation can be defined in unfractionated white blood cells. To date, three studies assessing genome-wide DNA methylation in Caucasian populations using the 450k microarray have been conducted to study the epigenetic abnormalities in specific blood cell lineages. Absher et al. investigated the genome-wide DNA methylation changes in CD4+ T cells, CD19+ B cells and CD14+ monocytes [20], whereas Coit et al. first studied the DNA methylation alterations in naïve CD4+ T cells [21] and furthered this analysis in neutrophils [22]. Though different blood cell lineages were studied, the same epigenetic signature showing hypomethylation in interferon genes were identified. Here, we demonstrated that fractionation of different white blood cells was not required to visualize this pattern of DNA methylation changes. In fact, among the 36 differentially hypomethylated CpG sites identified in our study, 35 of them were also reported in at least one of these three studies. This finding further suggests that the hypomethylated CpG sites identified in our study are the commonly altered CpG sites in SLE patients, regardless of ethnicity and blood cell subtype. The limitation of studying unfractionated white blood cells is that lineage-specific DNA methylation changes cannot be revealed; however, DNA from unfractionated white blood cells can be collected more easily, as only small volume of blood is needed, which is more acceptable to the recruited patients.

The third new finding we contribute is that we found an interesting pattern with respect to the genomic location of DNA hypomethylation. CpG sites showing loss of methylation were usually not located in a CpG island but rather in a CpG shore, which is the region approximately 0–2 kb away from a CpG island. Early discoveries demonstrated that DNA methylation changes can be associated with human disease through changes in DNA methylation at sites located either in gene promoters or CpG islands only [43, 44]. Then, a study of colon cancer revealed that functional changes occurred in CpG shores as well. It was also found that CpG shores, rather than CpG islands, are associated with most tissue-specific differential DNA methylation [45]. Since then, DNA methylation changes that occur in CpG shores have been identified in other diseases, such as breast cancer, hepatocellular carcinoma and type 2 diabetes. In fact, it is now evident that more CpG sites showing aberrant changes in DNA methylation are located in CpG shores than in CpG islands [46–48]. Our finding also suggests the functional importance of DNA methylation in CpG shores. We also looked at the DNA methylation data of different normal tissues obtained from the ENCODE project using the UCSC Genome Browser and found that in general, differentially methylated CpG sites located in CpG shores showed a distinct DNA methylation level between different tissues, whereas differentially methylated CpG sites located in regions other than CpG shores showed more or less the same DNA methylation level among different tissues (S9 Fig). The tissue-specific DNA methylation pattern located in CpG shores suggests its biological potential in determining tissue-specific functions, and loss of the original DNA methylation status in specific tissues could indicate the diseased stage of the cell.

Among the hypomethylated genes discovered in SLE patients, more and more attention is now focusing on *IRF7*. *IRF7* belongs to the family of interferon regulatory factors, encoding interferon regulatory factor 7. The interferon regulatory factors are well-known transcriptional regulators of type I interferon and the type I interferon-inducible genes [49]. Studies in a murine model demonstrated that *IRF7* is an essential master regulator for the production of type I interferon [50]. *IRF7* is activated by interaction with the adaptors *MyD88* and *TRAF6*, which in turn results in activation of the interferon-alpha gene promoter [50–52]. Furthermore, various genome-wide studies from different ethnic groups identified genetic variations in and around the coding region of *IRF7* that are associated with SLE [53, 54].

*IFI44L* is also another important gene that exhibits DNA methylation loss. It is known that type I interferon stimulates the expression of *IFI44L*, though the function of *IFI44L* remains unknown [55]. However, numerous studies found that *IFI44L* was hypomethylated in SLE patients [20–22], and recently Zhao et al. proposed that *IFI44L* promoter methylation can be used as a biomarker for distinguishing patients with SLE from healthy controls, and also other autoimmune diseases such as primary Sjögren’s syndrome and rheumatoid arthritis [56]. These results show that epigenetic analysis also provides opportunities for identifying novel biomarkers in SLE, and other epigenetic mechanisms, including histone modifications, 5-hydroxymethylcytosine and microRNA, have recently been reported to be dysregulated in SLE patients [57, 58]. These studies show that epigenetic factors, in addition to genetic factors, also play a role in SLE pathogenesis.

## Conclusion

In summary, our study of DNA methylation in unfractionated white blood cells demonstrated that in Chinese SLE patients from Hong Kong, differential DNA hypomethylation replicates the DNA methylation changes in Caucasian patients from whom fractionated white blood cells were tested for DNA methylation. DNA methylation changes occurred in gene-specific regions, especially CpG shores, and the genes were over-represented in type I interferon pathways, thus supporting the importance of type I interferon dysregulation in SLE pathogenesis.

## Supporting Information

S1 Fig Sequences flanking the differentially methylated CpG sites. The sequences flanking the differentially methylated CpG sites in *MX1*, *IFI44L*, *PLSCR1* and *NLRC5* are shown. All the differentially methylated CpG sites identified by the 450k microarray within the transcription start site of these genes are indicated, and the corresponding CG nucleotides in the sequence are underlined and highlighted in red.
(TIF)

S2 Fig Boxplot showing the average beta of control and SLE patients. There are a total of 48,578 probes on the 450k microarray, and 10 controls and 12 SLE patients were included for the downstream analysis. The box plot of the average beta for both the control group and SLE group is shown. There is no significant difference between control and SLE patients.
(TIF)

S3 Fig Hypermethylated genes found in SLE patients. Differentially hypermethylated genes are shown in the histogram, with the average beta of individuals of control and SLE patients.
(TIF)

S4 Fig Effect of blood cell type composition on the SLE DNA methylation signature. The DNA methylation signature of 12 SLE patients and 10 controls overlapping the 44 differentially methylated CpG sites was compared to the DNA methylation status at the same CpG sites from 48 different blood cell types from Reinius et al. Principal component analysis showed the distribution of these samples based on their DNA methylation profiles, in which all normal blood samples, including purified blood subtypes, were well separated from the SLE samples.
(TIF)

S5 Fig Methylation level of *MX1* and *NLRC5*. In addition to the differentially methylated CpG site detected by the microarray study, bisulfite pyrosequencing was also used to measure the DNA methylation level of CpG sites surrounding the differentially methylated one. (A) Boxplot showing the DNA methylation level of 7 CpG sites of *MX1*. The differentially methylated CpG site detected by microarray is CpG1. (B) Boxplot showing the DNA methylation level of 7 CpG sites of *NLRC5*. The differentially methylated CpG site detected by microarray is CpG7.
(TIF)

S6 Fig Effect of DNA methylation on mRNA expression of *MX1*, *IFI44L*, *PLSCR1* and *NLRC5*. Red spots refer to the data obtained from SLE patients, whereas blue spots refer to the data obtained from controls. The effect of DNA methylation of the differentially hypomethylated CpG sites identified in the microarray study on mRNA expression was studied, and it was demonstrated that mRNA expression was inversely correlated with DNA methylation level in *MX1*, *IFI44L*, *PLSCR1* and *NLRC5*.
(TIF)

S7 Fig Effect of the methylation of CpG sites on *MX1* mRNA expression. Red spots refer to the data obtained from SLE patients, whereas blue spots refer to the data obtained from controls. CpG1 is the differentially hypomethylated CpG site identified in the microarray study, whereas CpG2-CpG7 are the CpG sites surrounding CpG1. The data reveal that the methylation of sites CpG2-CpG7 correlate with mRNA expression more or less to the same extent as methylation of CpG1.
(TIF)

S8 Fig Effect of the methylation of CpG sites on *NLCR5* mRNA expression. Red spots refer to the data obtained from SLE patients, whereas blue spots refer to the data obtained from controls. CpG7 is the differentially hypomethylated CpG site identified in the microarray study, whereas CpG1-CpG6 are the CpG sites surrounding CpG1. The data reveal that the methylation of CpG1- CpG6 correlate differently to mRNA expression as compared to methylation of CpG1.
(TIF)

S9 Fig 450k microarray data collected by the UCSC Genome Browser. The 450k DNA methylation data of different normal tissues were searched in ENCODE. In general, CpG sites located in CpG shores showed a more variable DNA methylation level in different tissues compared to those located in regions other than CpG shores. Only hypomethylated genes detected in our 450k microarray study and those CpG sites with relations to CpG islands are shown.
(TIF)

S1 Table Clinical information of the 12 SLE patients participating in the microarray study. (DOCX)

S2 Table Bisulfite pyrosequencing assay design. (DOCX)

S3 Table Differentially methylated CpG sites identified in our study. (DOCX)
