# Supplementary figures and images for: Genome-Wide DNA Methylation Analysis of Chinese Patients with Systemic Lupus Erythematosus Identified Hypomethylation in Genes Related to the Type I Interferon Pathway
Source: PLoS One. 2017 Jan 13;12(1):e0169553. doi: 10.1371/journal.pone.0169553 (PMC5234836; doi:10.1371/journal.pone.0169553)

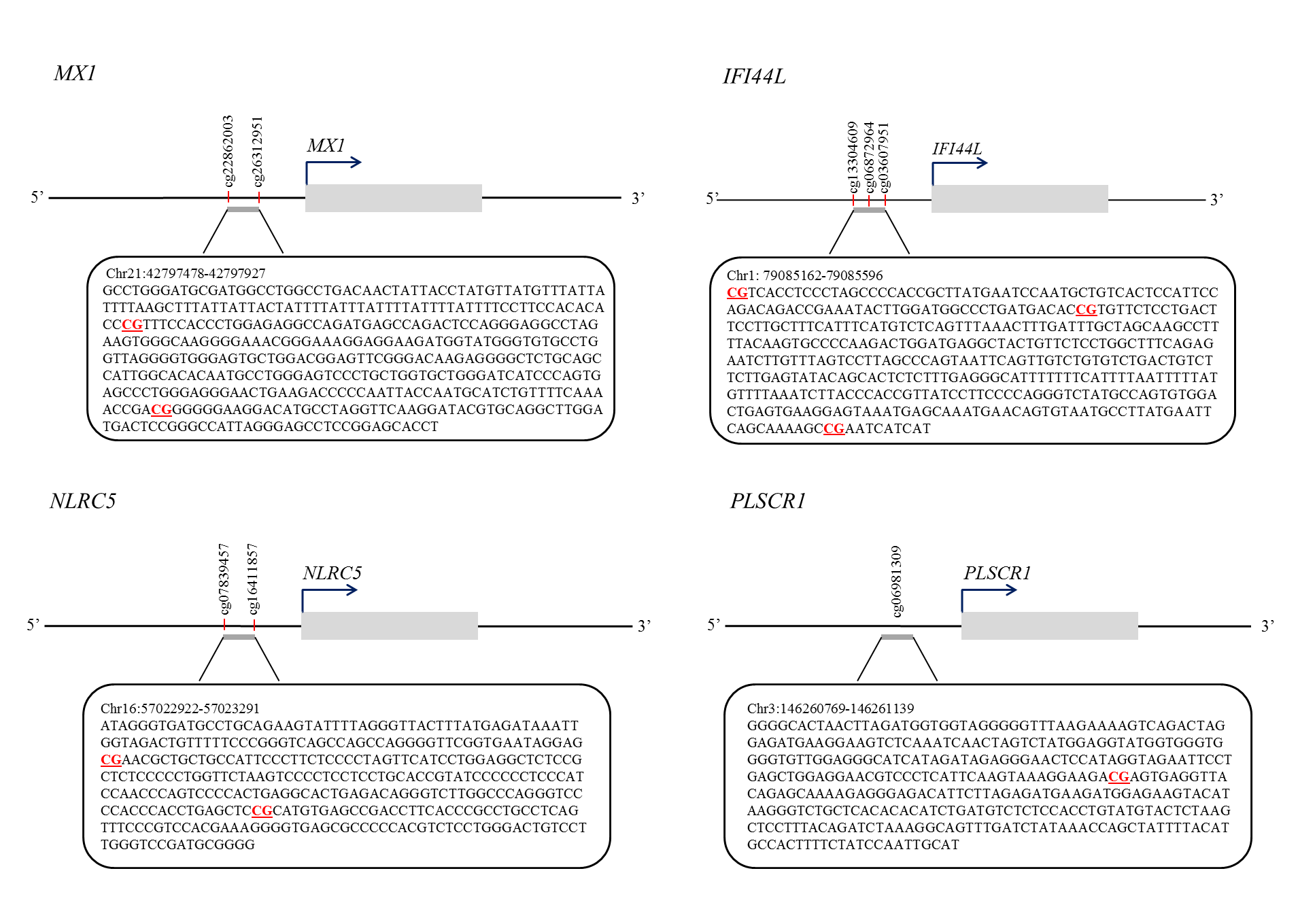

Supplement: S1 Fig — The sequences flanking the differentially methylated CpG sites in MX1, IFI44L, PLSCR1 and NLRC5 are shown. All the differentially methylated CpG sites identified by the 450k microarray within the transcription start site of these genes are indicated, and the corresponding CG nucleotides in the sequence are underlined and highlighted in red. (TIF) [file pone.0169553.s001.tif]

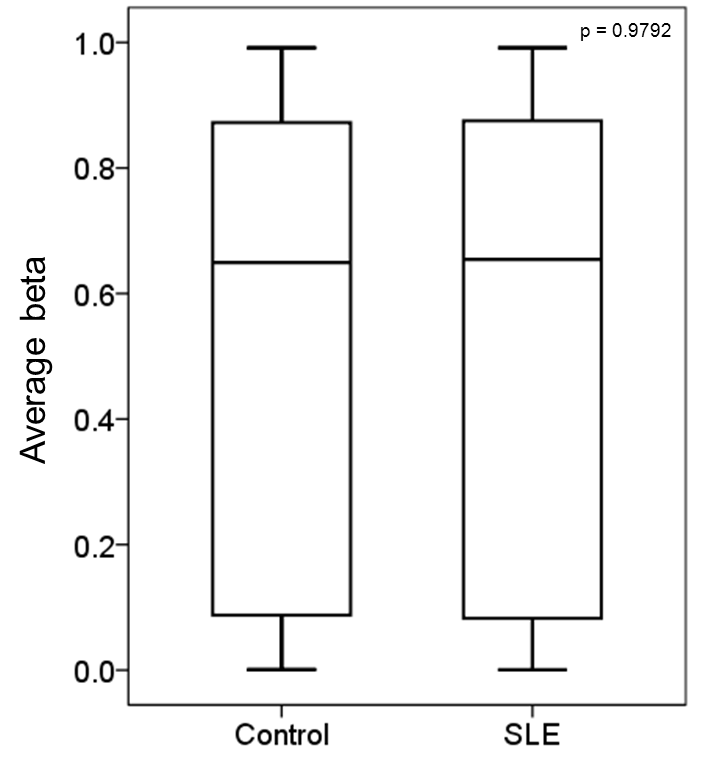

Supplement: S2 Fig — There are a total of 48,578 probes on the 450k microarray, and 10 controls and 12 SLE patients were included for the downstream analysis. The box plot of the average beta for both the control group and SLE group is shown. There is no significant difference between control and SLE patients. (TIF) [file pone.0169553.s002.tif]

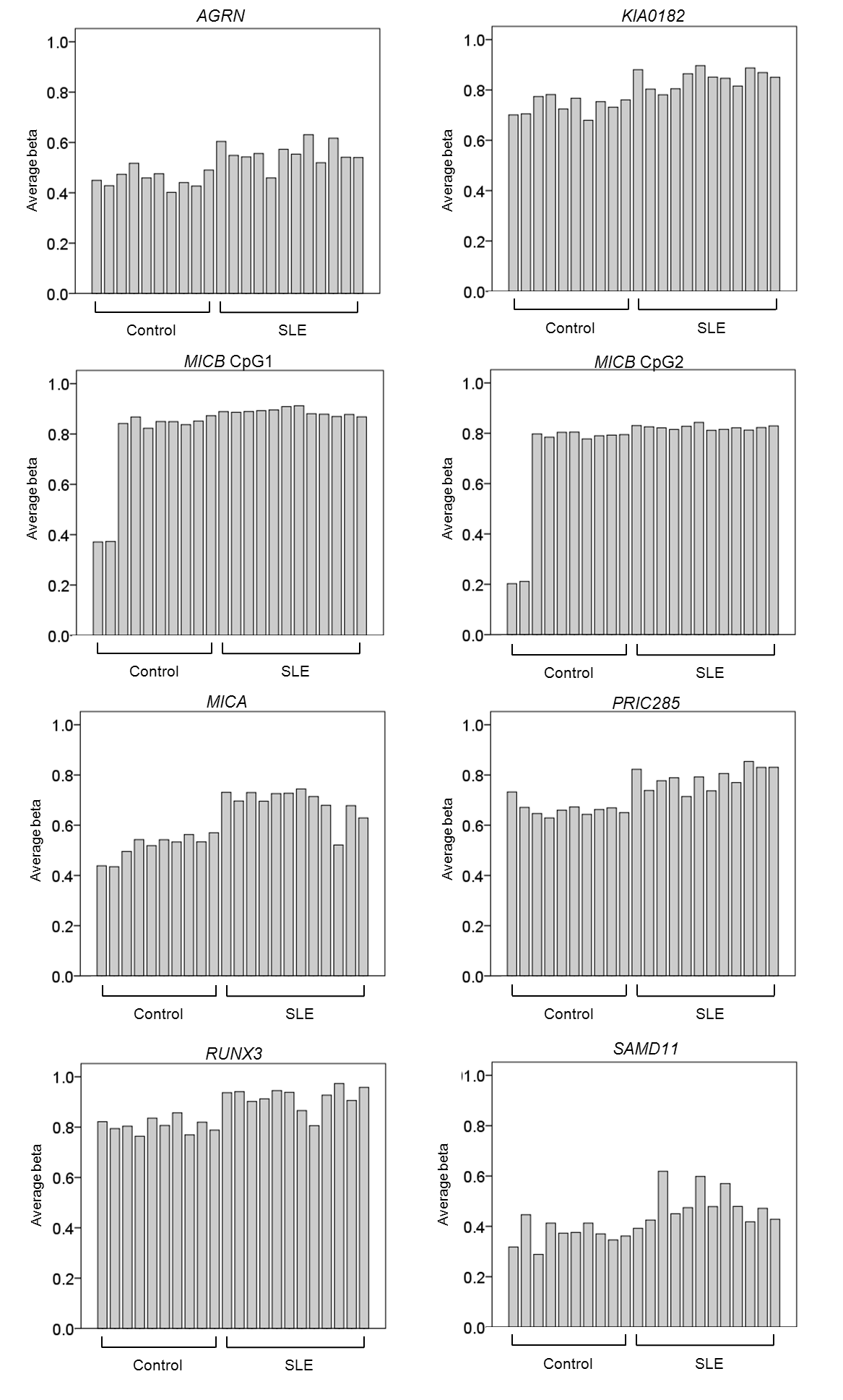

Supplement: S3 Fig — Differentially hypermethylated genes are shown in the histogram, with the average beta of individuals of control and SLE patients. (TIF) [file pone.0169553.s003.tif]

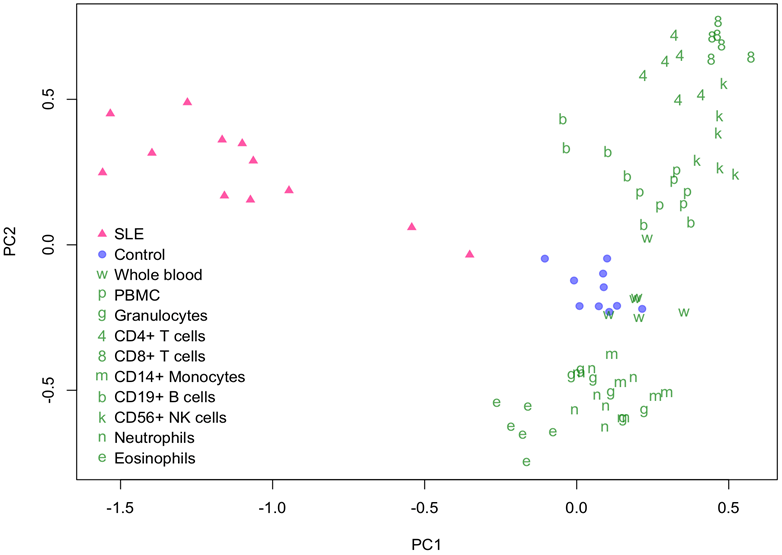

Supplement: S4 Fig — The DNA methylation signature of 12 SLE patients and 10 controls overlapping the 44 differentially methylated CpG sites was compared to the DNA methylation status at the same CpG sites from 48 different blood cell types from Reinius et al. Principal component analysis showed the distribution of these samples based on their DNA methylation profiles, in which all normal blood samples, including purified blood subtypes, were well separated from the SLE samples. (TIF) [file pone.0169553.s004.tif]

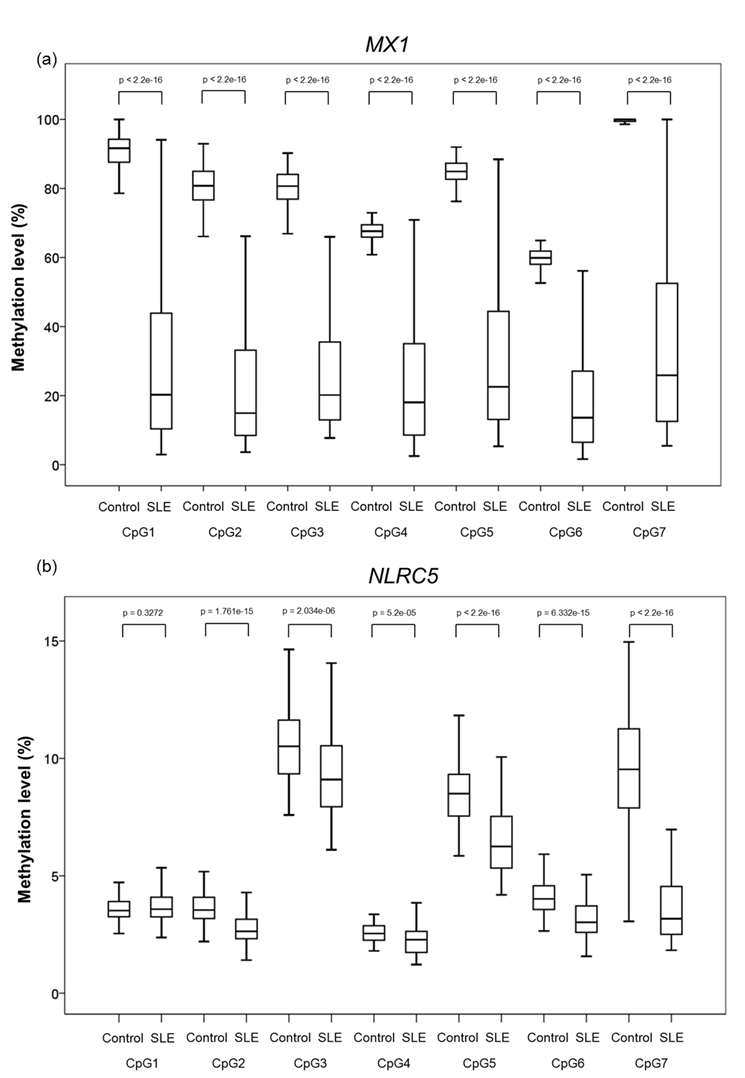

Supplement: S5 Fig — In addition to the differentially methylated CpG site detected by the microarray study, bisulfite pyrosequencing was also used to measure the DNA methylation level of CpG sites surrounding the differentially methylated one. (A) Boxplot showing the DNA methylation level of 7 CpG sites of MX1. The differentially methylated CpG site detected by microarray is CpG1. (B) Boxplot showing the DNA methylation level of 7 CpG sites of NLRC5. The differentially methylated CpG site detected by microarray is CpG7. (TIF) [file pone.0169553.s005.tif]

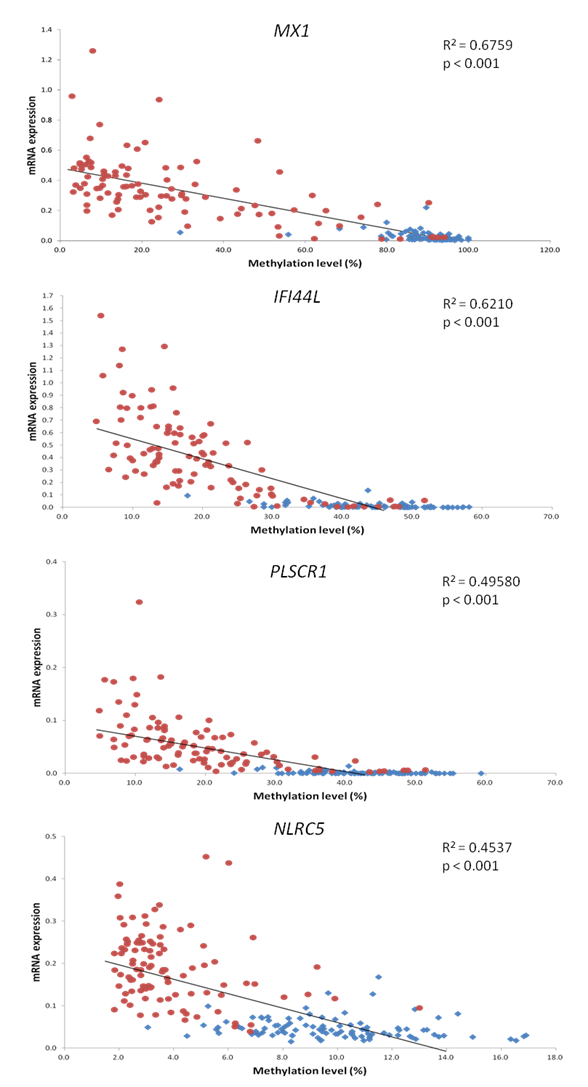

Supplement: S6 Fig — Red spots refer to the data obtained from SLE patients, whereas blue spots refer to the data obtained from controls. The effect of DNA methylation of the differentially hypomethylated CpG sites identified in the microarray study on mRNA expression was studied, and it was demonstrated that mRNA expression was inversely correlated with DNA methylation level in MX1, IFI44L, PLSCR1 and NLRC5. (TIF) [file pone.0169553.s006.tif]

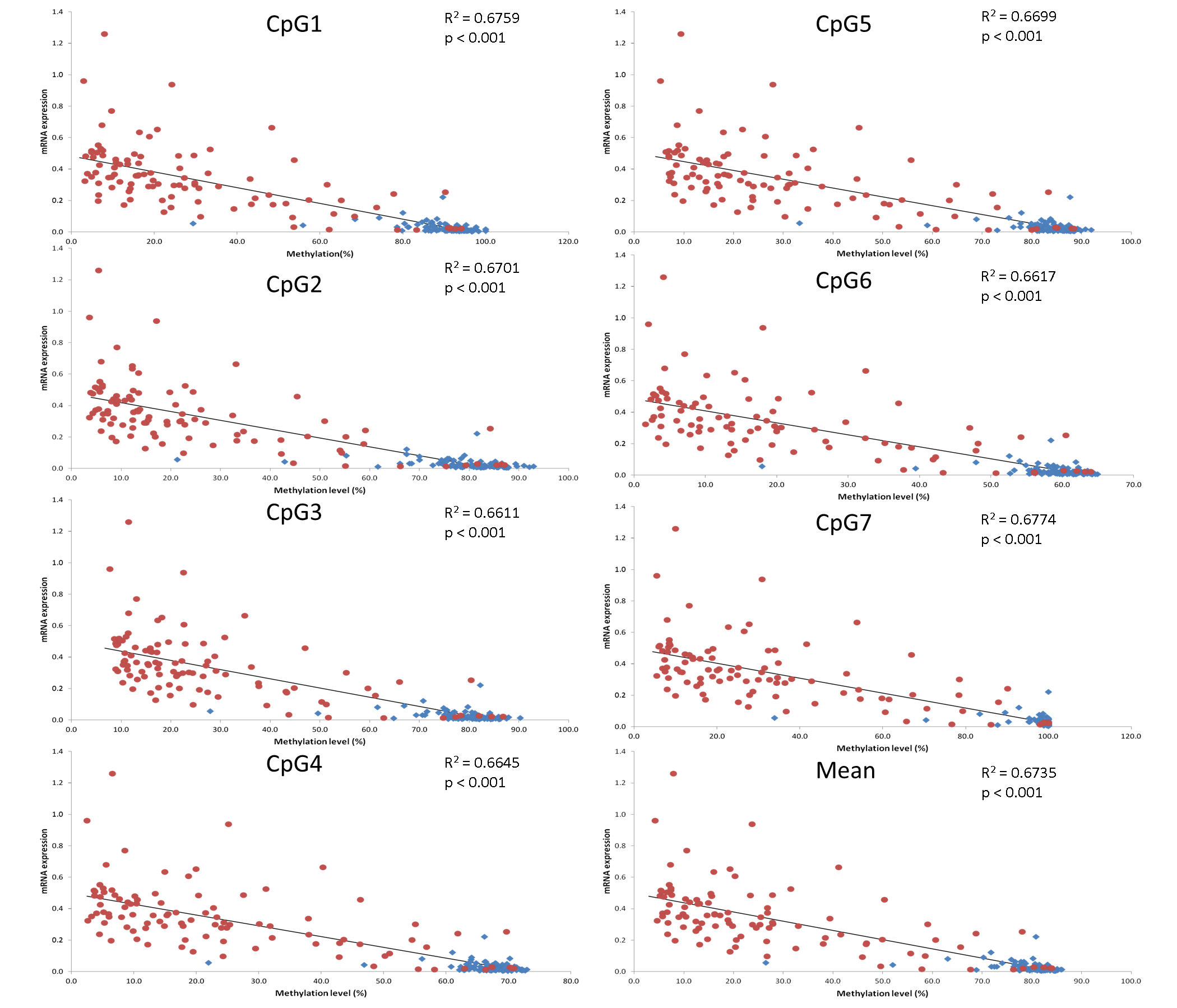

Supplement: S7 Fig — Red spots refer to the data obtained from SLE patients, whereas blue spots refer to the data obtained from controls. CpG1 is the differentially hypomethylated CpG site identified in the microarray study, whereas CpG2-CpG7 are the CpG sites surrounding CpG1. The data reveal that the methylation of sites CpG2-CpG7 correlate with mRNA expression more or less to the same extent as methylation of CpG1. (TIF) [file pone.0169553.s007.tif]

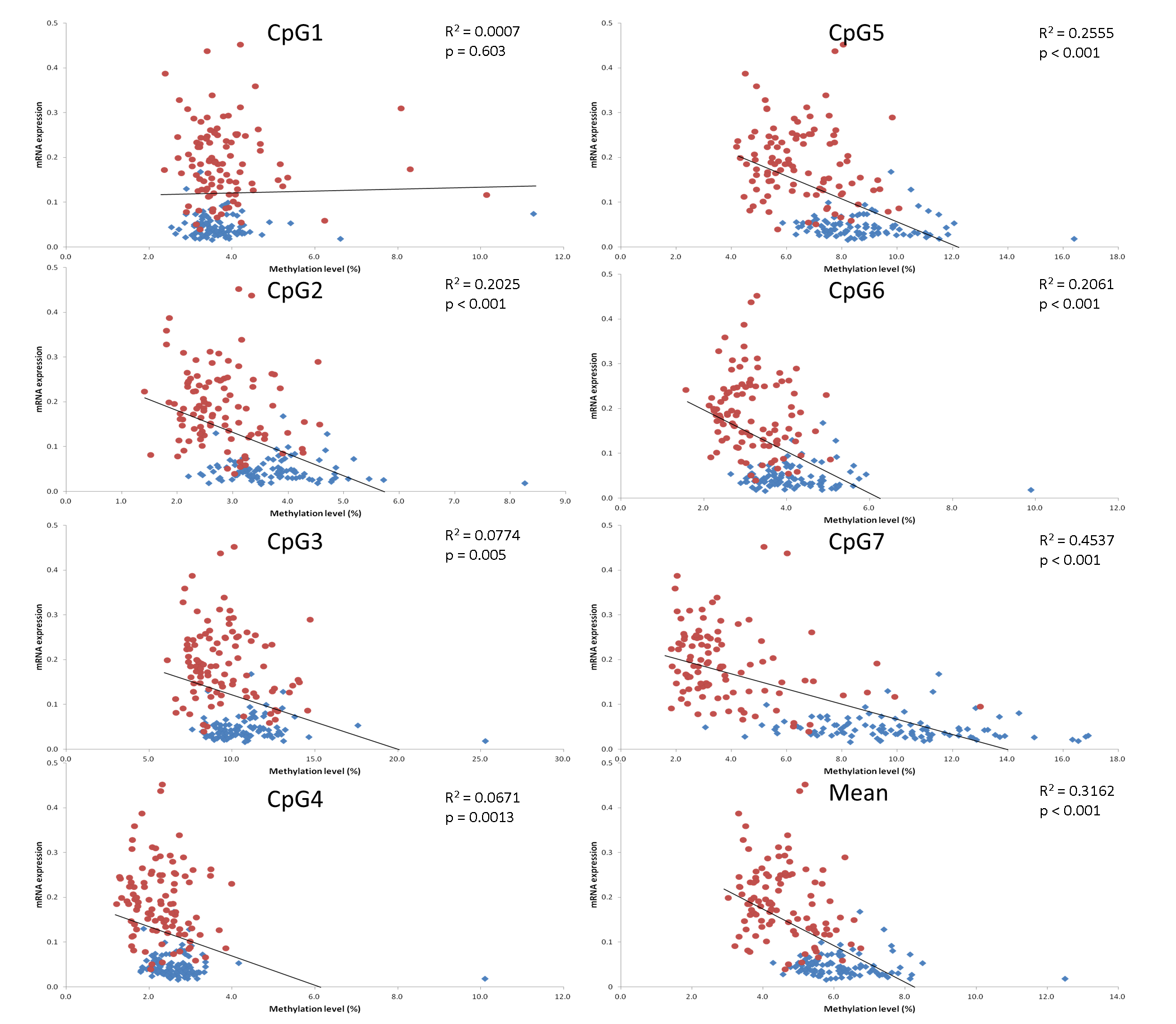

Supplement: S8 Fig — Red spots refer to the data obtained from SLE patients, whereas blue spots refer to the data obtained from controls. CpG7 is the differentially hypomethylated CpG site identified in the microarray study, whereas CpG1-CpG6 are the CpG sites surrounding CpG1. The data reveal that the methylation of CpG1- CpG6 correlate differently to mRNA expression as compared to methylation of CpG1. (TIF) [file pone.0169553.s008.tif]

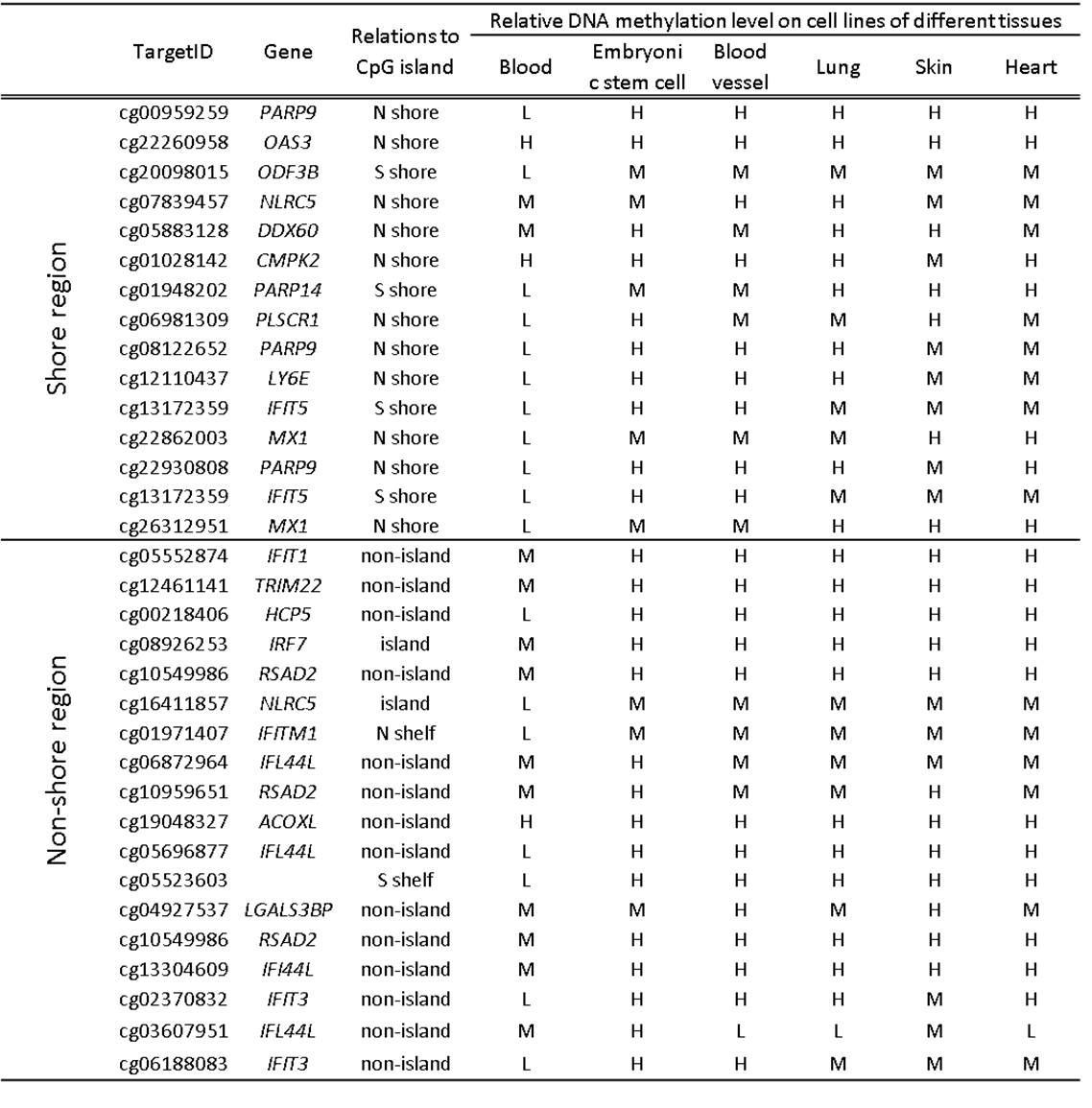

Supplement: S9 Fig — The 450k DNA methylation data of different normal tissues were searched in ENCODE. In general, CpG sites located in CpG shores showed a more variable DNA methylation level in different tissues compared to those located in regions other than CpG shores. Only hypomethylated genes detected in our 450k microarray study and those CpG sites with relations to CpG islands are shown. (TIF) [file pone.0169553.s009.tif]

**
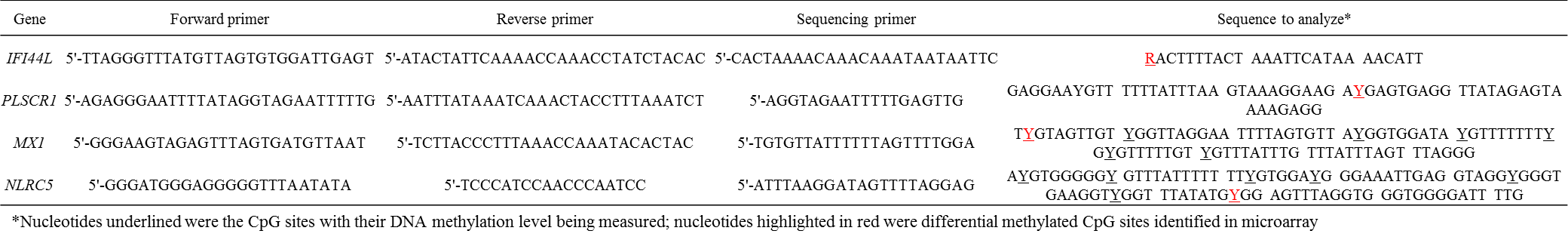
**

**TableS2: Assay design of bisulfite pyrosequencing**

Supplement: S2 Table — (DOCX) [file pone.0169553.s011.docx]
