# Supplementary material for: Genome-Wide DNA Methylation Analysis of Chinese Patients with Systemic Lupus Erythematosus Identified Hypomethylation in Genes Related to the Type I Interferon Pathway
Source: PLoS One. 2017 Jan 13;12(1):e0169553. doi: 10.1371/journal.pone.0169553 (PMC5234836; doi:10.1371/journal.pone.0169553)
Supplement: S1 Table — (DOCX) [file pone.0169553.s010.docx]

| Patient | Onset age | Age of DNA  sampling | SLEDAI  score | Clinical and biological features related to SLE | *Medication (per day) |
| --- | --- | --- | --- | --- | --- |
| 1 | 20 | 23 | 10 | Proteinuria, alopecia, low C3&C4, increased DNA binding | P6, MMF 650mg |
| 2 | 30 | 34 | 0 | Nil | P 10mg, MMF 500mg |
| 3 | 16 | 19 | 2 | Increased DNA binding | P 5mg, aza 25mg, HCQ 200mg |
| 4 | 27 | 29 | 4 | Low C3&C4, leukopenia | P 4mg, HCQ 200mg |
| 5 | 33 | 34 | 4 | New rash, low C3, thrombocytopenia | HCQ 200mg |
| 6 | 24 | 26 | 4 | Low C3, increased DNA binding | P 7.5mg, MMF 1000mg |
| 7 | 19 | 23 | 2 | Low C3 | P 5mg, HCQ 200mg, MMF 500mg |
| 8 | 37 | 40 | 4 | Proteinuria | P 5mg |
| 9 | 25 | 29 | 4 | New rash, low C3 | P 7.5 mg, HCQ 200mg, MMF 1000mg |
| 10 | 17 | 19 | 4 | New rash, low C3 | P 5mg, aza 100mg |
| 11 | 25 | 27 | 2 | Low C3 | P 5mg, HCQ 200mg |
| 12 | 41 | 42 | 4 | Proteinuria | P 10mg |

*P= Prednisolone; A= Azathioprine; HCQ = Hydroxychloroquine sulphate; MMF= mycophenolate mofetil

**TableS1: Clinical information of 12 SLE patients participating in microarray study**
