# Supplementary material for: Genome-Wide DNA Methylation Analysis of Chinese Patients with Systemic Lupus Erythematosus Identified Hypomethylation in Genes Related to the Type I Interferon Pathway
Source: PLoS One. 2017 Jan 13;12(1):e0169553. doi: 10.1371/journal.pone.0169553 (PMC5234836; doi:10.1371/journal.pone.0169553)
Supplement: S3 Table — (DOCX) [file pone.0169553.s012.docx]

| No* | TargetID | Mean Beta | | Beta  Difference | Adjusted  p-value | UCSC reference  gene name | UCSC  reference  gene group | Relations to  CpG island | Reported by | | |
| --- | --- | --- | --- | --- | --- | --- | --- | --- | --- | --- | --- |
|  |  | Control | SLE |  |  |  |  |  | Coit^1^ | Absher^2^ | Coit^3^ |
| 1 | cg05696877 | 0.5896 | 0.2076 | -0.3820 | 0.0194 | *IFI44L* | 5'UTR |  | ✓ | ✓ | ✓ |
| 2 | cg22930808 | 0.6644 | 0.3003 | -0.3641 | 0.0154 | *PARP9;DTX3L* | 5'UTR;TSS1500 | N_Shore | ✓ | ✓ | ✓ |
| 3 | cg00959259 | 0.5230 | 0.1888 | -0.3342 | 0.0194 | *PARP9;DTX3L* | 5'UTR;TSS1500 | N_Shore |  | ✓ |  |
| 4 | cg08122652 | 0.7730 | 0.4698 | -0.3032 | 0.0230 | *PARP9;DTX3L* | 5'UTR;TSS1500 | N_Shore | ✓ | ✓ | ✓ |
| 5 | cg22862003 | 0.6991 | 0.4037 | -0.2953 | 0.0154 | *MX1* | TSS1500;5'UTR | N_Shore | ✓ | ✓ | ✓ |
| †6 | cg03607951 | 0.5067 | 0.2297 | -0.2770 | 0.0154 | *IFI44L* | TSS1500 |  | ✓ | ✓ | ✓ |
| 7 | cg07839457 | 0.4835 | 0.2130 | -0.2706 | 0.0154 | *NLRC5* | TSS1500 | N_Shore | ✓ | ✓ | ✓ |
| 8 | cg05552874 | 0.6577 | 0.3936 | -0.2641 | 0.0273 | *IFIT1* | Body |  | ✓ | ✓ | ✓ |
| 9 | cg06872964 | 0.4867 | 0.2448 | -0.2419 | 0.0194 | *IFI44L* | TSS1500 |  | ✓ | ✓ | ✓ |
| †10 | cg26312951 | 0.4125 | 0.1797 | -0.2328 | 0.0230 | *MX1* | TSS200;5'UTR | N_Shore | ✓ | ✓ | ✓ |
| 11 | cg13172359 | 0.4826 | 0.2614 | -0.2212 | 0.0318 | *IFIT5* | Body | S_Shore |  | ✓ |  |
| 12 | cg12110437 | 0.5070 | 0.2944 | -0.2126 | 0.0273 | *LY6E;LOC100133669* | TSS1500;Body | N_Shore | ✓ | ✓ | ✓ |
| 13 | cg06188083 | 0.5137 | 0.3058 | -0.2079 | 0.0318 | *IFIT3* | Body |  | ✓ | ✓ | ✓ |
| 14 | cg20045320 | 0.5967 | 0.3912 | -0.2055 | 0.0377 |  |  | S_Shore |  | ✓ | ✓ |
| †15 | cg06981309 | 0.4575 | 0.2565 | -0.2009 | 0.0154 | *PLSCR1* | 5'UTR | N_Shore | ✓ | ✓ |  |
| 16 | cg01028142 | 0.8194 | 0.6188 | -0.2007 | 0.0230 | *CMPK2* | Body | N_Shore | ✓ | ✓ | ✓ |
| 17 | cg13304609 | 0.8192 | 0.6277 | -0.1915 | 0.0194 | *IFI44L* | TSS1500 |  |  | ✓ | ✓ |
| 18 | cg05883128 | 0.4804 | 0.2934 | -0.1869 | 0.0318 | *DDX60* | 5'UTR | N_Shore | ✓ | ✓ | ✓ |
| 19 | cg01948202 | 0.2519 | 0.0787 | -0.1732 | 0.0154 | *PARP14* | Body | S_Shore | ✓ | ✓ | ✓ |
| 20 | cg20098015 | 0.4732 | 0.3204 | -0.1528 | 0.0154 | *ODF3B* | TSS200 | S_Shore |  | ✓ | ✓ |
| 21 | cg02370832 | 0.6624 | 0.5100 | -0.1523 | 0.0230 | *IFIT3* | Body |  |  | ✓ |  |
| 22 | cg05523603 | 0.6685 | 0.5167 | -0.1518 | 0.0377 |  |  | S_Shelf |  | ✓ |  |
| 23 | cg08926253 | 0.5891 | 0.4397 | -0.1494 | 0.0318 | *IRF7* | Body | Island |  | ✓ | ✓ |
| 24 | cg04927537 | 0.4748 | 0.3259 | -0.1489 | 0.0273 | *LGALS3BP* | TSS200 |  |  |  | ✓ |
| 25 | cg10549986 | 0.1982 | 0.0538 | -0.1444 | 0.0154 | *RSAD2* | 1stExon |  | ✓ | ✓ | ✓ |
| 26 | cg13130398 | 0.8540 | 0.7246 | -0.1295 | 0.0438 | *RABGAP1L* | TSS1500;Body |  |  |  | ✓ |
| 27 | cg10959651 | 0.2806 | 0.1527 | -0.1279 | 0.0230 | *RSAD2* | 1stExon |  | ✓ | ✓ | ✓ |
| 28 | cg01971407 | 0.5084 | 0.3866 | -0.1218 | 0.0438 | *IFITM1* | TSS1500 | N_Shelf |  | ✓ | ✓ |
| 29 | cg19048327 | 0.8343 | 0.7145 | -0.1198 | 0.0318 | *ACOXL* | 3'UTR;Body |  |  | ✓ |  |
| 30 | cg08574632 | 0.6801 | 0.5607 | -0.1194 | 0.0194 |  |  |  |  |  |  |
| 31 | cg12461141 | 0.5157 | 0.4056 | -0.1101 | 0.0154 | *TRIM22* | TSS1500 |  |  | ✓ |  |
| 32 | cg22260958 | 0.8383 | 0.7284 | -0.1099 | 0.0230 | *OAS3* | TSS1500 | N_Shore |  |  |  |
| 33 | cg00218406 | 0.3884 | 0.2803 | -0.1080 | 0.0438 | *HCP5* | 3'UTR |  |  | ✓ |  |
| 34 | cg14870271 | 0.3984 | 0.2904 | -0.1080 | 0.0230 | *LGALS3BP* | 1stExon;5'UTR |  |  | ✓ |  |
| 35 | cg22764925 | 0.6573 | 0.5528 | -0.1045 | 0.0273 | *GGT1* | 5'UTR |  |  | ✓ | ✓ |
| †36 | cg16411857 | 0.2056 | 0.1019 | -0.1037 | 0.0230 | *NLRC5* | TSS1500 | Island |  | ✓ | ✓ |
| 37 | cg13100600 | 0.4558 | 0.5582 | 0.1024 | 0.0318 | *AGRN* | Body | S_Shelf |  |  |  |
| 38 | cg26657404 | 0.7406 | 0.8504 | 0.1098 | 0.0194 | *KIAA0182* | Body | S_Shore | ⬝ | ⬝ |  |
| 39 | cg05162523 | 0.8090 | 0.9230 | 0.1140 | 0.0377 | *RUNX3* | Body |  | ⬝ | ⬝ |  |
| 40 | cg13692836 | 0.3670 | 0.4835 | 0.1165 | 0.0438 | *SAMD11* | Body | Island |  | ⬝ |  |
| 41 | cg23352030 | 0.6655 | 0.7934 | 0.1279 | 0.0194 | *PRIC285* | Body;1stExon | Island | ⬝ | ⬝ |  |
| 42 | cg17510601 | 0.7535 | 0.8873 | 0.1338 | 0.0212 | *MICB* | Body | S_Shelf | ⬝ | ⬝ |  |
| 43 | cg01101177 | 0.6730 | 0.8298 | 0.1568 | 0.0154 | *MICB* | Body | S_Shelf | ⬝ | ⬝ |  |
| 44 | cg27610521 | 0.5176 | 0.6975 | 0.1798 | 0.0438 | *MICA* | Body | S_Shelf | ⬝ | ⬝ |  |

* Prioritized according to accending beta difference
†probes chosen for bisulfite pyrosequencing


1Coit P, Jeffries M, Altorok N, Dozmorov MG, Koelsch KA, Wren JD, Merrill JT, McCune WJ, Sawalha AH. Genome-wide DNA methylation study suggests epigenetic accessibility and transcriptional poising of interferon-regulated genes in naive CD4+ T cells from lupus patients. Journal of autoimmunity 2013; 43:78-84.

2Absher DM, Li X, Waite LL, Gibson A, Roberts K, Edberg J, Chatham WW, Kimberly RP. Genome-wide DNA methylation analysis of systemic lupus erythematosus reveals persistent hypomethylation of interferon genes and compositional changes to CD4+ T-cell populations. PLoS genetics 2013; 9:e1003678.

3Coit P, Yalavarthi S, Ognenovski M, Zhao W, Hasni S, Wren JD, Kaplan MJ, Sawalha AH. Epigenome profiling reveals significant DNA demethylation of interferon signature genes in lupus neutrophils. Journal of autoimmunity 2015; 58:59-66.

**TableS3: Differential methylated CpG sites identified in our study**
